# Modelling and Analysis of Central Metabolism Operating Regulatory Interactions in Salt Stress Conditions in a L-Carnitine Overproducing *E. coli* Strain

**DOI:** 10.1371/journal.pone.0034533

**Published:** 2012-04-13

**Authors:** Guido Santos, José A. Hormiga, Paula Arense, Manuel Cánovas, Néstor V. Torres

**Affiliations:** 1 Departamento de Bioquímica y Biología Molecular, Universidad de La Laguna, San Cristóbal de La Laguna, Santa Cruz de Tenerife, Spain; 2 Departamento de Bioquímica y Biología Molecular B, Universidad de Murcia, Murcia, Spain; University of Westminster, United Kingdom

## Abstract

Based on experimental data from *E. coli* cultures, we have devised a mathematical model in the GMA-power law formalism that describes the central and L-carnitine metabolism in and between two steady states, non-osmotic and hyperosmotic (0.3 M NaCl). A key feature of this model is the introduction of type of kinetic order, the osmotic stress kinetic orders (g_OSn_), derived from the power law general formalism, which represent the effect of osmotic stress in each metabolic process of the model.

By considering the values of the g_OSn_ linked to each metabolic process we found that osmotic stress has a positive and determinant influence on the increase in flux in energetic metabolism (glycolysis); L-carnitine biosynthesis production; the transformation/excretion of Acetyl-CoA into acetate and ethanol; the input flux of peptone into the cell; the anabolic use of pyruvate and biomass decomposition. In contrast, we found that although the osmotic stress has an inhibitory effect on the transformation flux from the glycolytic end products (pyruvate) to Acetyl-CoA, this inhibition is counteracted by other effects (the increase in pyruvate concentration) to the extent that the whole flux increases. In the same vein, the down regulation exerted by osmotic stress on fumarate uptake and its oxidation and the production and export of lactate and pyruvate are reversed by other factors up to the point that the first increased and the second remained unchanged.

The model analysis shows that in osmotic conditions the energy and fermentation pathways undergo substantial rearrangement. This is illustrated by the observation that the increase in the fermentation fluxes is not connected with fluxes towards the tricaboxylic acid intermediates and the synthesis of biomass. The osmotic stress associated with these fluxes reflects these changes. All these observations support that the responses to salt stress observed in *E. coli* might be conserved in halophiles.

Flux evolution during osmotic adaptations showed a hyperbolic (increasing or decreasing) pattern except in the case of peptone and fumarate uptake by the cell, which initially decreased. Finally, the model also throws light on the role of L-carnitine as osmoprotectant.

## Introduction

Stress responses and their underlying mechanisms are of foremost interest not only because they are critical for determining cell physiology and metabolism in such conditions but also because they reveal some conserved features that are largely independent of the organism [1].

Extensive investigation into *E. coli* stress responses [2]–[5] has shown that major components of the general and specific response regulate key cellular processes. However, most of the studies on the *E. coli* response to osmotic stress environmental changes refer to the transcription level. Despite some analyses [6]–[10], the mechanisms involved in the adaptation of the central and energy metabolism of bacteria to osmotic conditions remain largely unstudied. Important in this regard is the fact that, contrary to most cases where signaling is involved, the osmoregulation signals which eventually translates into changes in metabolism and physiology, are of a physicochemical rather that of chemical nature [11]. This paper deals with the osmoregulation of *E. coli* at the metabolic level; that is, the changes in metabolic processes which, prompted by signals associated with the new osmolarity conditions, occur during adaptation to increasingly saline environments.

Important in this regard are some aspects that greatly determine the scope of our study. First, attention should be drawn to the distinction between the osmoregulatory mechanisms acting in the long term (when the system has reached a steady state in constant osmolarity conditions), and short term mechanisms, of a transient nature that occur in changing osmolarity conditions. Interestingly, little information is available concerning the long-term effects of salt stress exposure on the central metabolism of *E. coli*. In addition, in the case of hyperosmotic stress in *E. coli*, one well observed homeostatic mechanism is the synthesis of some osmoprotectants, non-toxic molecules that help compensate the osmotic pressure by accumulating in the intracellular medium. These osmoprotectants include K^+^, amino acids such as glutamate and proline, and trehalose and zwitterionic organic solutes like L-carnitine, [12], [13] a trimethylammonium compound well described as osmoprotectant in this microorganism [14], [15]. Further, experimental evidence on the important role played by salt stress on the energetic state and the main metabolic pathways of the cells during metabolic biotransformations of trimethylammonium compounds in *E. coli* has been shown [6].

Starting with the results of an anaerobic culture of a L-carnitine *E. coli* overproducing strain subjected to an osmotic stress [10], we have constructed a mathematical model which reproduces both the non-osmotic and hyperosmotic steady states, as well as the observed dynamic behavior of the transition between them. Our aim was to quantify the signals and observed changes in fluxes; in particular, we wished to contribute to the elucidation and quantification of the response generated in *E. coli* by hyperosmotic stress as regards the primary metabolism (central carbon) and its relationship with the synthesis of an osmoprotectant, L-carnitine, produced by a secondary metabolism.

## Materials and Methods

### Experimental Methods

The experimental set up and data used to construct and verify the model were taken from [10]. In the experimental set up used by these authors, an L-carnitine overproducer *E. coli* (O44K74AS) strain adapted to high salt concentration was used. After removal of the storage medium, the bioreactor was operated in the presence of this strain and a standard complex medium at a fixed dilution rate. Once the initial steady state under standard salt conditions (control, 0.085 M NaCl) was reached, a gradual osmotic up-shift was carried out by feeding the culture with a medium containing 0.3 or 0.5 M NaCl until a new, hyperosmotic medium steady state was reached. This new steady state was reached 70 h after switching the medium. This time period of 70 hours can be considered a long-term *E. coli* cell adaptation period [10], [16] since at least 12 generations were produced to keep the cell population steady within the bioreactor. This number of cell divisions meant that many changes and protein turn-overs, the result of gene expressions and adaptations, had occurred to reach the steady state within the reactor at the new salt concentration [10]. The osmotic up-shift was carried out when the culture had reached the steady state.

### Mathematical modeling

The approach used to model this biochemical system involves mathematical models in ordinary differential equations in the power-law formalism. In this formalism [17] the processes that conforms the biochemical networks are modelled using power-law expansions in the variables of the system and are then included in non-linear ordinary differential equations with the following structure, called Generalized Mass Action (GMA):
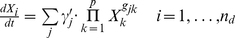
(1)In Equation 1, *X_i_* represents the model variables (concentrations of compounds of the investigated network: metabolites, salt medium, bacterial concentration, etc.), *n_d_* is the number of model variables, and *γ'_j_* (rate constants) and *g_jk_* (kinetic orders) are different kinds of parameters defining the dynamics of the system. Unlike in conventional kinetic models, where the kinetic orders are always integer numbers, power-law models admit non-integer values.

In order to deal with the description and quantification of the effect of the osmotic stress in the metabolic processes we use a new type of kinetic order, the osmotic stress kinetic orders (g_OSn_), derived from the power law general formalism. In the definition of this system parameter, we follow a similar approach to that presented by Fonseca et al. [18] for the study and quantification of the temperature dependence of the system. While these authors introduce a parameter that represents the change in enzymatic activity brought about by an increase in temperature, our kinetic orders (g_OSn_) represent the change caused by the osmotic stress.

In our case, the rate constants γ'_j_ can be expressed as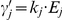, where *k_j_* depends on the properties of the molecular medium, such as the pH, temperature or salt concentration (in our case only the salt, 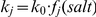 and E_j_ is the enzyme concentration. The enzyme concentration, E_j_, under stress conditions also changes, 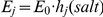. Accordingly, 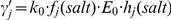. In the phenomenological approach here proposed the effect of osmotic stress in the rate constant and the enzyme activity is aggregated into a single characteristic power-law term:
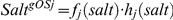
Therefore Equation 1 in the osmotic stress conditions under consideration becomes:
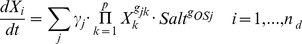
(2)The most notable property of these power-law equations is that two such equations with the same structure can be used to describe totally different dynamics (from inhibitory processes to the description of cooperativity) simply by modifying the numerical values of the kinetic orders involved [19]. Moreover, this type of representation has been shown to be suitable and effective for the modelling of dynamic signalling structures. It has been shown that virtually any linear or non-linear system can be represented accurately by a GMA of higher dimension through algebraic equivalence transformations of variables in a process called recasting [20].

#### Delay definition

A time-delay was applied to the model to represent the different time scale between some distinct qualitative processes within the cell (cell death, gene expression, etc.). This delay was modeled using the linear chain trick [21], whereby the delay was described using time-dependent fiction variables (in our case, *Delay_name_*_1_ and *Delay_name_*_2_, where *name* is the name of the variable to which the delay is applied). The features of distributed time-delay (average value and standard deviation) depend on the number of fictitious variables used and the value assigned to the rate constant *K_delay_*. We noticed that more sophisticated strategies are available to introduce time delay in ordinary differential equation models (see for example, [22]). However, our initial analysis indicated that the simple strategy used to model time-delay was sufficient for our case study.

#### Parameter estimation

The numerical values for the parameters *γ*_i_ and *g_jk_* are determined using an evolutionary algorithm called simple genetic algorithm (SGA) adapted to determine the values of parameters in power law models [23], [24]. This algorithm starts with a population of individuals, which are the set of parameters that generates a solution of the model. The population is mutated and recombined; the convergence stems from minimization of the value of the objective function (*Fobj*):
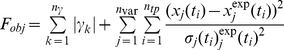
(3)In Equation 3, 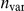
and 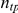 are the model variables and time points, respectively; n_γ_ is the number of rate constants of the model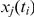 and 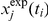
are the value of variable *j* at time *i* based on the simulation of the model and the experimental data of the variable j at time *i*, respectively; 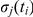 is the standard deviation of the experimental value of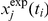. Furthermore, an additional term in Equation 3 was introduced in order to obtain a more restrictive objective function. This term is the sum of the γ's, in absolute values, which allow the value of these parameters in the solutions to be reduced. γ is associated with the enzyme concentration; the enzyme concentration (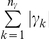) is minimized in the data fitting process.

The initial point integrating for the variables should be at the same time an experimental and analytical steady state value. The last condition is fulfilled by a set of system parameters satisfying equations 3.
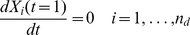
(4)In the search for a solution, SGA was run through 10,000 iterations using the 0.3 M salt concentration time series data. Initially, a first set of *γ_j_* and *g_jk_* parameters was found within the range searched (*γ_j_* = [0, 20]; *g_jk_* = [−3, 3]), showing a low value of *Fobj*. Then, the previously obtained parameter solution was used in the subsequent searches within a 95% of the first solution, the *Fobj* between 25% and 60% of the initial *Fobj* in all cases.

#### Stability analysis

All eligible solutions should show initial and final stable steady states. The stability of the steady states was checked by analyzing the behavior of the model against small perturbations.

#### Dynamic sensitivity analysis

Sensitivity analysis is a general analysis tool useful for evaluating robustness and characterizing system dynamics. Since our model studies the system dynamics, it is possible to identify those parameters with a major influence on the transient dynamics. For this purpose we used a modified version of previously defined dynamic sensitivity [24], the System Dynamic Sensitivity, S*_pk_* ^Xi^, as indicated in equation 5:
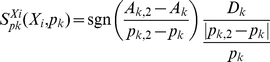
(5)In the above expression, Xi is the considered variable, while *pk* is the parameter under scrutiny. A*k,2* is the area below the solution curve, after a change of *pk* to *pk,2*. A*k* is the area under the solution curve using the original value *pk*. D*k* is the area between the two solution curves, using *pk* and *pk,2*, respectively. We have considered changes in two kinds of parameter, the kinetic orders and the rate constants. The S*_pk_* ^Xi^ value corresponds to the variation of the area under the variable time course after perturbation in parameter space. For each model variable the absolute values of the area *A_k_*, *A_k2_* and *D_k_* were calculated using the trapezoidal method. Positive sensitivity means that the area under the ÒnewÓ curve is greater than the area under the original curve i.e. that the parameter *pk* has a positive influence on variable Xi. Negative sensitivity means the opposite. Zero sensitivity means that small changes in the parameter have no influence on the variable. All sensitivities were computed for standardized variables.

### Kinetic processes

**Figure 1:**
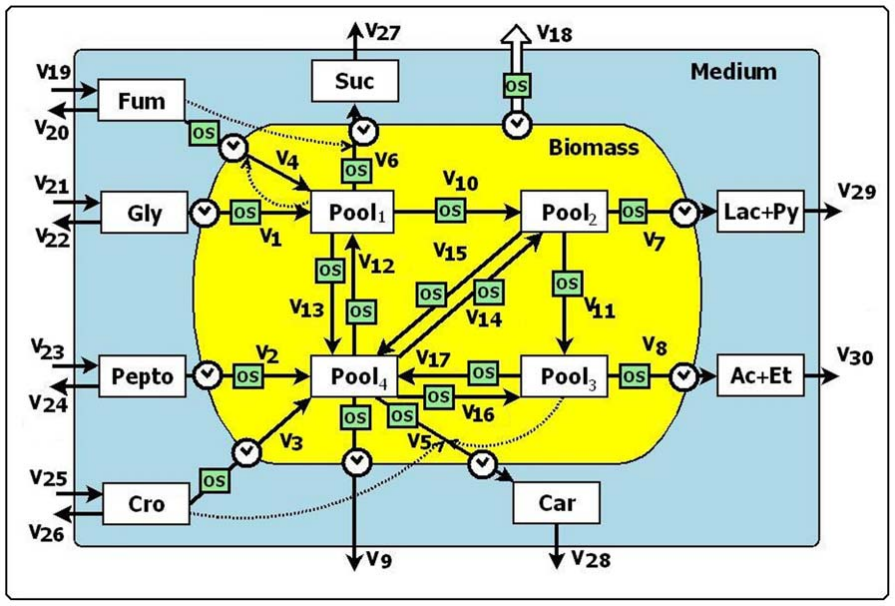
Schematic representation of the central metabolism of a L-carnitine overproducing *E. coli* strain applicable both to normal and osmotic stress conditions. Solid lines represent rate processes and dotted arrows represent regulatory interactions. The empty arrow represents the output flux of biomass. The “clock” symbols represent a time-delay in a process whose value is a parameter estimated during the model calibration. The OS boxes represent the influence of the osmotic stress on the flux rate. See text for detail.

Figure 1 shows the simplified version of the central metabolism of *E. coli* applicable both to normal and osmotic stress conditions where the intracellular variables are connected to the external measured ones.

Since our objective here is to analyze the effect that changes in the salt concentration of the medium provoke in the dynamics of the system, we focused our attention on the main central metabolism fermentation processes. We also pay attention to the mass flux looking at the metabolite accumulation and biomass production and the flux exchanges among the cell cultures, the medium and the bioreactor. Accordingly, four variables aggregate all the intracellular compounds. As is shown in Figure 1, three pools (Pool_1_, Pool_2_ and Pool_3_) connect the carbon source (glycerol, Gly) with the fermentation products lactate and pyruvate (Lac+Py) and acetate and ethanol (Ac+Et) as well as with the fumarate (Fum) reduction into succinate (Suc). Pool_4_, on its own, aggregates the rest of the components of the cell and it is connected with the rest of the Pools. Presented in this way the model is simple enough to be defined and to be user friendly, but still provide information on the influence of the osmotic stress on the metabolic rearrangement.

More specifically, Pool_1_ represents the intracellular concentration of phosphoenolpyruvate, succinate, fumarate, malate and oxaloacetate; Pool_2_ includes pyruvate and lactate; Pool_3_, acetyl CoA, acetyl-P, acetate, acetaldehyde and ethanol, while Pool_4_ is a variable representing the rest of the intracellular cell compounds; among them, the rest of the intermediates of the tricarboxilyc acid cycle. Biomass, which is not directly represented in Figure 1, where the yellow area represents the variable biomass as the sum of all the variables within it, is an auxiliary variable, defined as the summation of the four intracellular Pool_i_ variables. Thus defined, Biomass serves to represent the mass of microorganism in the reactor. Biomass is defined as the whole population in the reactor, and it is measured as the total cell dry weight (mg)/ml of the reactor bulk liquid. During the different cell divisions biomass adapts to the new steady state after the salt up-shift [10], [25]. Therefore, the individual cell content was adapted to the new conditions and can be represented by the summation of the Pools, and the total amount of cells can be represented by the total cell dry weight (mg)/ml of the reactor bulk liquid. Since the biomass changes during the transition, fluxes were normalized to the biomass unit, so that they are referred to milligrams of cell mass.

The reactor medium and the extracellular metabolite concentrations are represented by Lac+Py, the combined concentrations of lactate and pyruvate; Ac+Et is the summation of pyruvate, acetate and ethanol; Gly, is the glycerol concentration, and Pepto represents the concentration of peptone.

#### Quantification of direct and indirect influence of osmotic stress on metabolism

In this model the influence of osmotic stress conditions on the flux values is represented by of the power law term Salt^gOSj^ (indicated as OS, green boxes in Figure 1). In this expression Salt is the normalized salt concentration (NaCl) of the medium with respect to the control conditions (0.085 M). Salt quantifies the osmotic stress, being 1 in control conditions, 3.53 in 0.3 M NaCl conditions and 5.88 in 0.5 M.

The exponential terms g_OSj_ are the osmotic kinetic orders. These are phenomenological parameters, that quantify the combined effect on the fluxes (V_j_) of the mediating mechanisms between the medium salt concentration (the osmotic stress) and the fluxes. These mechanisms are of a physicochemical and transcriptional (changes in enzyme concentrations) nature. The term g_OSj_ can have positive or negative values, reflecting an increase or decrease of the flux due to the osmotic stress. This modelling strategy permits us to separately quantify the influence of osmotic stress (through the g_OSj_) and of the metabolite concentration (g_jk_) on the fluxes

#### Transport and central metabolic processes

The central metabolism and the glycerol and peptone uptake by cells are also depicted in Figure 1. V_1_ is the glycolytic flux (glycerol to phosphoenolpyruvate, indicate by Pool_1_); V_10_ the transformation from Pool_1_ (mainly phosphoenolpyruvate) to Pool_2_ (pyruvate) through pyruvate kinase, and, V_11_ is the transformation from Pool_2_, namely pyruvate, into Pool_3_ (acetyl CoA) through pyruvate dehydrogenase. Fluxes V_12_, V_14_ and V_16_ are catabolic fluxes mediated by enzymes: V_12_ is associated with the phosphoenolpyruvate carboxykinase activity and also represents the thehalose degradation processes that yield pyruvate from any other precursor, particularly the TCA intermediates and the amino acid degradation; V_14_ represents the synthesis of pyruvate from other metabolites. V_16_ represents different processes leading to the synthesis of Pool_3_ from precursors other than glycerol (lipids and/or proteins and L-carnitine). V_13_, V_15_ and V_17_ are anabolic processes. V_13_ is associated with the intracellular production of trehalose, the net flux between Pool_1_ and Krebs cycle intermediates, the PEP-carboxylase activity and the transformation of these intermediates into Biomass [10]; V_15_ represents the transformation of the pyruvate component of Pool_2_ into some precursors (amino acids and osmoprotectants). V_17_ is related with the flux through citrate synthase, including glutamate biosynthesis. V_7_ is the combined transformation/excretion of pyruvate and lactate (Pool_2_), which is mediated by the reference enzyme lactate dehydrogenase, while V_8_ is the combined transformation/excretion of Pool_3_ into acetate and ethanol (Ac+Et) mediated by the reference enzymes Acetyl-CoA synthetase and Phosphotransacetylase. V_2_ is the input flux of peptone into the cell and V_9_ is the biomass output flux (for instance in the form of NH_4_^+^ and/or CO_2_ release).

#### Fumarate reduction and succinate production

Fumarate (Fum) is antiported with intracellular succinate and it is not used as carbon source [26] . Once fumarate is within the cell, it is reduced and transformed (V_4_) into internal succinate (Pool_1_), which eventually leaves the cell, V_6_. The positive regulatory influence of fumarate on V_6_ collects its role as substrate in the production of succinate [10].

#### Bioreactor system

The whole system (open and continuous) has overall input and output fluxes. V_19_ to V_30_ represent the influxes and the outflows of the corresponding variables, all of which are mediated by the dilution rate (K_d_ = 0.1 h^−1^). V_18_ (empty arrow) represents the output of biomass from the reactor.

#### L-carnitine transport, production and degradation

Cell crotonobetaine (Cro) uptake flux from the reactor medium is represented by V_3_; V_5_ is the transformation/output flux of L-carnitine from the cell to the reactor medium (Car). V_5_ is linked to the variables Pool_3_ and Cro through activating regulatory interactions in accordance [14].

### Model Equations

In accordance with the model scheme shown in Figure 1, the system model equations are as follows.

#### Mass Balance

The mass balance equations of the processes take the following form:
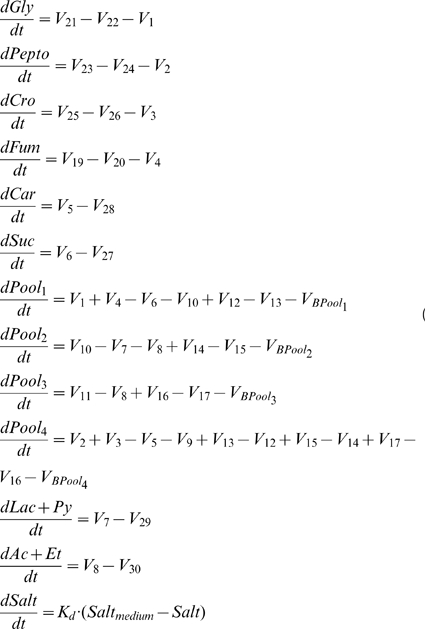
(6)As said above, biomass is the sum of Pool_i_ (i = 1, 2, 3, 4). In Figure 1, V_18_ represents the output of biomass, but since biomass is a variable composed of four terms, V_18_ is the sum of four biomass contributions coming from each one of the internal variables of Pool_i_, namely V_BPool1_, V_BPool2_, V_BPool3_ and V_BPool4_. Accordingly, these fluxes are represented in the corresponding Pool_i_ differential equations (see Equations 6).

Salt_medium_ is the normalized concentration of salt with respect to the control condition in the input medium to the reactor and Salt as defined above.

#### Delay equations

The delay equations that describe the longer temporal hierarchies of the enzyme expression processes are modelled using a three-equation linear chain trick. Delay is applied to the variable Biomass, which is the sum of the variables of the model Pool_1_, Pool_2_, Pool_3_ and Pool_4_, so this delay is applied to each of these variables as a proportional part of the biomass that they conform (Equations 6).
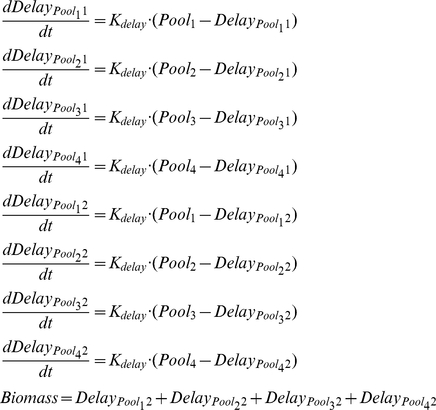
(7)


#### Power law rate equations

The rate equations in power law form corresponding to the different fluxes are:
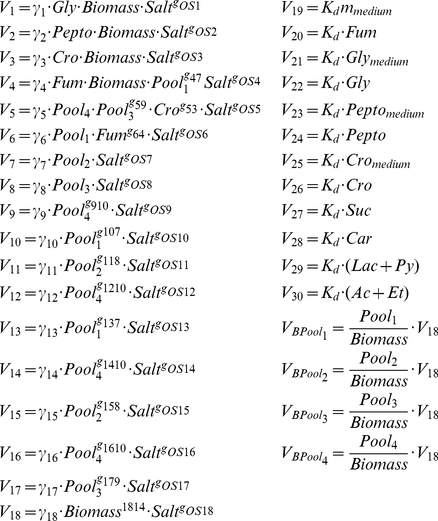
(8)


#### System parameters and constants

In this model approach the rate constants (γ_j_) are associated with the net enzyme activity of each process and are considered to remain constant. It should be noted here that since the osmotic kinetic orders measures the effect on the fluxes of the mechanisms that mediate between the osmotic stress and the fluxes, they include in their values the effects on the fluxes associated with the changes that occurs in enzymes (concentration, effectors, coenzymes etc.). Kinetic orders (g_jk_) measure the effect of each variable on the flux V_j_. Gly_medium_, Peptone_medium_, Cro_medium_ and Fum_medium_ are the concentration of glycerol, peptone, crotonobetaine and fumarate in the input medium to the reactor, respectively, which are kept constant.

#### Identifiability of solutions

Many set of parameters showing similar values of the objective function were found. In order to select the most representative ones, a selection criterion was applied. The selection criterion used ensures that the fitting of each variable is independent from the remaining variables. Since the objective function can give solutions with a poor fitting in one of the variables if another variable has a very good fit, the criterion mentioned above eliminate these kinds of solutions. According with this criterion only was admitted as solutions for the osmotic steady state (0.3 M) those in which the values of the dependent variables were within the range ±1.5 SD of the experimental values of osmotic steady state, except in the case of Lac+Py and Car (±3 SD) and Biomass (±4.5 SD). The value of the ranges was estimated from the standard deviation of the data.

#### Model validation

The model was validated using a new set of data which were not used in the parameter fitting procedure. This data were obtained at 0.5 M salt concentration. Solutions obtained by fitting with the 0.3 M data were tested against the experimental data obtained at 0.5 M NaCl. In these conditions the only solutions admitted were those in which the dependent variables were within the range ±3 SD of the experimental values. In the case of the variables Lac+Py and Car and Biomass the range was ±6 SD and ±9 SD, respectively.

## Results and Discussion

### Model fitting and verification

**Figure 2:**
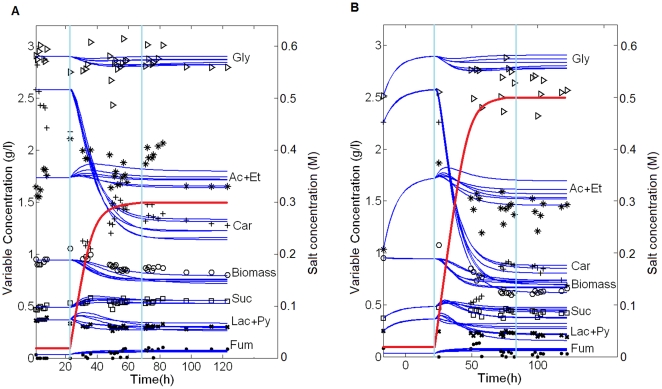
Model fitting and verification. In all panels continuous lines represent the six best model solutions are represented (they are represented simultaneously in order to see the differences between them). The first vertical line indicates the moment (23 hours) when the medium switch begins by NaCl addition, while the second one indicates when the final steady state is reached (70 hours). Red lines represent the salt concentration in the extracellular medium. A. Model fitting in conditions of 0.3 M of final NaCl concentration. B. Model verification in conditions of 0.5 M of final NaCl.

Figure 2 shows the simultaneous integration of the six selected solutions obtained with the procedures described above; this representation shows the differences between them. Panel A shows the model data fitting in 0.3 M of NaCl conditions; panel B shows a comparison of the model predictions with the experimental time series data in 0.5 M of NaCl conditions. In all cases the first vertical line (23 hours) indicates the moment when salt switching begins (at the initial 0.085 M NaCl control steady state), and the second one (70 hours) the moment when the system reaches the final steady state (0.3 or 0.5 M NaCl, respectively). The mean values of the parameters of the six solutions as well as the whole set of parameter solutions for each of the solutions are shown in Tables S1 and S2 of Supporting Information files, respectively. The model fits the experimental data well (*Fobj* = 195.82±20.84) and is able to predict the general behavior in different, more saline conditions (0.5 M NaCl).

### Sensitivity and stability analysis

All solutions were in a stable steady state before and after the salt concentration switch. Variable sensitivities for parameter changes and initial conditions showed distinct pattern distribution for the different solutions considered. However, all sensitivity values were in the range [1.0825, −0.7377], 99% of all sensitivities being between −0.1 and 0.1 (data not included). This indicates that the selected solutions are robust for changes in parameters and initial conditions.

### Concentrations change in variables

**Figure 3:**
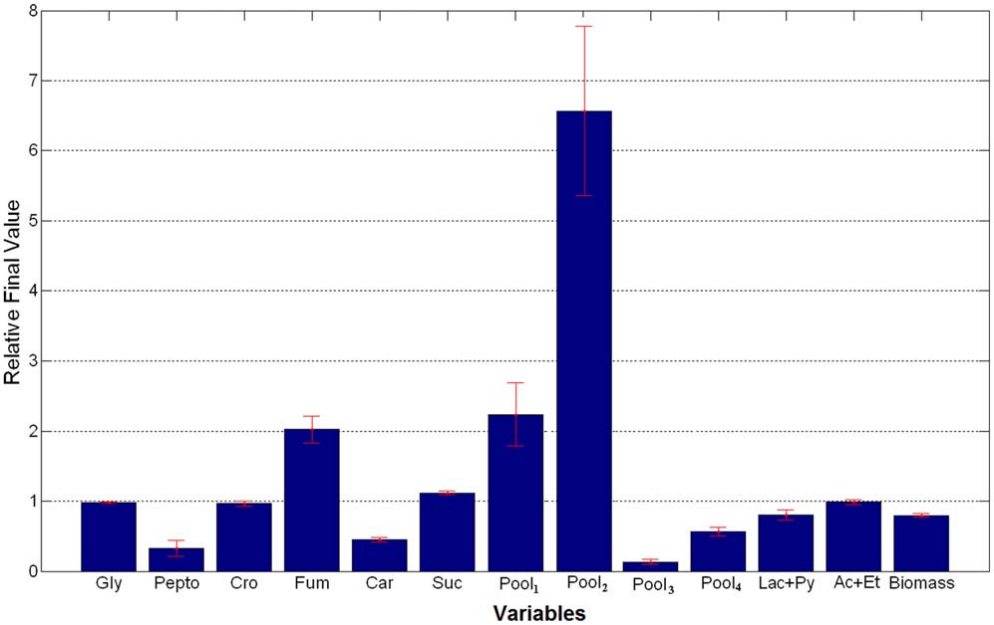
Relative variable concentration changes. Mean values of the variables concentration changes for the selected solutions between the final, osmotic stress (0.3 M NaCl) and the initial steady state. Values were calculated as the ratio between the final and the initial steady states. All values are normalized with respect to the initial steady state values.

Figure 3 shows the relative changes in variables concentrations between the final (0.3 M NaCl) and the initial steady state. It can be seen that the concentration of all the variables increased in osmotic conditions. In particular Pool_2_ increased more than 6 times with respect to its value in nonosmotic conditions and Pool_1_ and Fum doubled in concentration.

### Kinetic order distribution for metabolic changes and osmotic stress

In previous studies [6], [10] it was shown that, as a result of long-term exposure to NaCl, the metabolism of *E. coli* adapts to stress conditions, the adaptation being mediated by changes in enzyme activities and cofactor levels. Integration of the experimental data into a comprehensive model provides new evidences regarding the actual flux distribution.

**Figure 4:**
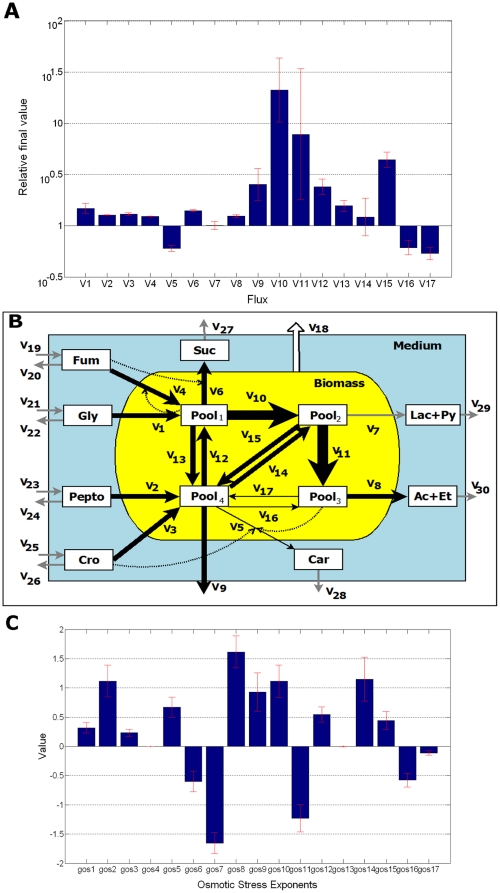
Flux changes and osmotic stress kinetic order distribution in osmotic conditions (0.3 M). **A.** Mean flux changes in logarithmic scale of (the mean of) the selected solutions. Relative change was calculated as the ratio between the flux values (normalized by the biomass and the flux at the initial steady state) in the osmotic steady state and the corresponding normalized flux in the initial steady state. **B.** The magnitude of the flux changes are indicated by the thickness arrows. Thicker arrows represent fluxes which increase their value in stress condition; thinner ones are the fluxes that decrease their value after the salt switch, while the gray ones correspond to unchanged fluxes. All fluxes are normalized to the concentration of the biomass. **C.** g_OS_ kinetic order value (mean values).

Figures 4A and 4B show the magnitude of the flux changes from different perspectives. It can be seen that the fluxes showing the increases in the 0.3 M steady state were V_10_ and V_11_. To a lesser degree, V_1_, V_2_, V_3_, V_4_, V_6_, V_8_, V_9_, V_12_, V_13_, V_14_ and V_15_, also decreased. Some fluxes (V_5_, V_16_ and V_17_) decreased when in osmotic conditions while V_7_ remained practically unchanged.

Figure 4C show the estimated g_OS_ kinetic orders. As stated above the value of these parameters measure the effect of osmotic stress on the corresponding fluxes. As can be seen, all of them are within the [−2, 2] range value. Those showing higher positive values, indicating a major positive influence of the osmotic conditions on the corresponding fluxes, are g_OS2_ (V_2_), g_OS8_ (V_8_), g_OS9_ (V_9_), g_OS10_ (V_10_) and g_OS14_ (V_14_). Also positive but of minor magnitude are g_OS1_ (V_1_), g_OS3_ (V_3_), g_OS5_ (V_5_), g_OS12_ (V_12_) and g_OS15_ (V_15_). On the other hand, the g_OS_ showing the greatest negative values are g_OS7_ (V_7_) and g_OS11_ (V_11_). Negative too, but less so, were: g_OS6_ (V_6_), g_OS16_ (V_16_) and g_OS17_ (V_17_). Parameters g_OS4_ (V_4_) and g_OS13_ (V_13_) have zero values.

As stated above, the influence of osmotic stress conditions on the flux values is represented by the term Salt^gOSn^. Examination of this term in the context of each flux rate equation (see Equations 7) allows us to analyse the magnitude and significance of the osmotic associated interactions on each of the fluxes. The main results of this section are summarized in Figure 4.

One observed change after the salt switch was the increase in the flux V_1_. This flux is dependent on Gly and Salt and represents the flux from external glycerol (Gly_medium_) to internal Pool_1_. The corresponding kinetic order g_OS1_ is 0.3, meaning that the osmotic stress has a moderate positive effect in the input of glycerol. Since variable Gly remains constant (see Figure 2), we conclude that the osmotic stress is responsible for this flux increase. This result shows that there is a mechanism which relates osmotic stress with the entrance and transformation of glycerol into Pool_1_.

Another result arises from the change in the fluxes V_10_ and V_11_. V_10_, which increase its value in osmotic conditions, represents the transformation from Pool_1_ to Pool_2_. Its value is dependent on Pool_1_ and Salt through the kinetic orders g_107_ (1.6) and g_OS10_ (1.1), respectively. Since both kinetic orders are positive and the values of Pool_1_ and Salt are higher in osmotic conditions (Figure 3) the positive influence of the osmotic stress must be added to the positive effect of the other variables. A different situation is observed in the case of V_11_, the flux representing the transformation from Pool_2_ to Pool_3_. V_11_ depends on Pool_2_ and Salt through the kinetic orders g_118_ (1.7) and g_OS11_ (−1.2), respectively. In this case, despite the negative value of g_OS11_, V_11_ increased, the positive effect of the combined Pool_2_ increased and its greater kinetic order g_118_ being enough to compensate the negative influence of Salt^gOS11^ term. It may be suggested that the increase in V_10_ is to meet the energy requirements, while the increase in V_11_ serves to compensate Pool_2_ accumulation. This would permit increased energy synthesis in order to resist the selective pressure imposed and would explain the increased rates of generation of fermentation products.

V_8_, the combined transformation/excretion of Pool_3_ into Ac+Et, reflects a different situation. This flux, which is dependent on Pool_3_ and Salt through the kinetic orders 1 and g_OS8_ (1.6), increases in osmotic conditions. In the stressed steady state, Pool_3_ decreases significantly (87%) although the strong and positive influence of Salt^gOS8^ causes this flux to increase its value. This behavior correlates with an increase in energy production as a result of substrate level phosphorylation under osmotic stress.

V_4_, the fumarate uptake, increases and the associated osmotic kinetic order (g_OS4_) is null. Thus, the osmotic stress does not affect V_4_. Since the other terms affecting V_4_ either decrease (Pool_1_^g47^; g_47_ being negative) or increases (Fum) the observed increase is due to the increase of Fum. In the same vein, V_6_, which represents Fum respiration and succinate output, increases its value. This increase in flux cannot be attributed to an osmotic stress mechanism since its influence is negative (g_OS6_ = −0.6) and must be attributed to the positive influence of the increase of Fum (g_64_ = 0.12) and Pool_1_.

V_15_, which represents the anabolic use of pyruvate, increase its value more than 4 times. In this case the osmotic stress kinetic order is positive (g_OS6_ = 0.44), as is the effect of Pool_2_, whose concentration increases more than 5 times (g_158_ = 0.37).

Flux V_9_ represents the loss of biomass in the form of CO_2_ and NH_4_^+^. This flux increases in osmotic conditions. Since the Pool_4_ value decreases, the increase in V_9_ arises from an osmotic stress response mechanism (g_OS9_ = 0.93).

V_12_, the flux through the phosphoenolpyruvate carboxykinase activity (also linked to the trehalose degradation), increases due to the osmotic stress (g_OS12_ = 0.54). Contrary to the observed increase in V_13_, a aggregated process, which is not influenced by the osmotic stress (g_OS13_ = 0).

Finally, V_7_, which integrates the production and export of lactate and pyruvate does not change. In this case, the osmotic stress has a negative effect (g_OS7_ = −1.65), but is compensated by the positive effect of Pool_2_.

### The role of osmoregulators

The effects of osmotic stress on different systems pertaining to many, if not all, biological kingdoms are essentially the same [11]. The main and most common mechanism observed in such systems, aiming to keep within the physiologically acceptable boundaries the intracellular milieu, consists of the accumulation of nontoxic compatible solutes in the cytoplasm. These compatible solutes (osmolytes) can be either produced by the cell or assimilated from the extracellular medium.

In many instances, the first event in osmotic stress adaptation is the uptake of K^+^. The increase in K^+^ concentration not only serves to decrease the osmotic pressure, but is a primer signal for the production of other compatible solutes. One of these is glutamate, whose accumulation quickly reflects the K^+^ concentration increase, thus helping to neutralize the increase of positive charges. In a second phase, these K^+^ and glutamate concentrations decrease (the first one through excretion and the second one through its metabolic transformation), while trehalose is synthesized in a process that usually marks the end of metabolic osmotic adaption.

In the assayed experimental system the culture medium contained crotonobetaine, which, after uptake, yields a potent osmoregulator, L-carnitine. In our model V_3_ represents the input of crotonobetaine to produce L-carnitine (Pool_4_). We observed that this flux increases by 30%, its related osmotic stress kinetic order being positive (g_OS3_ = 0.23). Since Cro remains unchanged, the single reason for the increase in crotonobetaine input is the salt stress response mechanism.

In our model the production of glutamate is represented by flux V_17_, which is dependent on Pool_3_ and Salt through the corresponding kinetic orders g_179_ (0.3) and g_OS17_ (−0.1), respectively (see Table S1; Supplementary Materials). After the salt switch, a decrease in this flux occurs, indicating that glutamate is not significantly synthesized in response to the salt rise. This suggests that there is, instead, an accumulation of other compatible solutes from the extracellular medium, such as the trimethylammonium compounds involved in the medium (Cro and Car).

L-carnitine metabolism is inhibited in osmotic stress conditions [10]. In our model, the catabolism of L-carnitine is represented by V_16_, while its synthesis and excretion are represented by V_5_. V_16_ is dependent on the variables Pool_4_ and Salt through the kinetic orders g_1610_ (null value) and g_OS16_ (−0.6), respectively. On its own V_5_ is dependent on the variables Pool_4_, Pool_3_, Cro and Salt through the kinetic orders 1, g_59_ (0.5), g_53_ (1.15) and g_OS17_ (0.6), respectively. Our results show that the magnitudes of these fluxes are reduced in osmotic conditions (see Figure 4B). In the case of V_16_, the determinant (negative) factor is the osmotic stress response since the g_1610_ value is null. But in the case of V_5_, the observed decrease in the flux seems to be due to the combined effects of the decrease in Pool_4_ and Pool_3_ in spite of the counteracting influence of the osmotic stress. This indicates that the carnitine metabolism is down regulated by an osmotic stress response mechanism, leading to the accumulation of the osmoprotectant L-carnitine, while its excretion is not affected by the osmotic stress.

Another effect revealed by our model refers to the metabolism of peptone, which is present in the reactor medium. Peptone is made up of peptides and amino acids, among them proline (46 mg/g peptone, MC24 bacteriological peptone from LAB M laboratory). It is known that proline uptake from the extracellular medium plays a role as osmoprotectant by eliciting the (rapid) excretion of K^+^ and contributing to the depletion of trehalose [11]. Some of our model observations clearly correlate with these observations. First, it can be seen that flux V_2_, which in our model represents peptone uptake, increases (27%) when in osmotic conditions, suggesting increased proline uptake. At the same time V_12_, which also represents trehalose catabolism, increases more than two-fold. The values of the related osmotic kinetic orders, g_OS2_ and g_OS12_, related with V_2_ and V_12_, respectively, have positive values (1.1 and 0.5, respectively). These results suggest that the input of peptone (V_2_) and trehalose catabolism (V_12_) are affected by the osmotic stress.

### Concluding remarks

In analyzing the influence of osmotic stress on flux redistribution in the osmotic stressed steady state, not only the regulatory effects inherently associated with the osmotic stress response mechanisms should be considered, but also the simultaneous influence of the changes that occur in other variables in the system. The model approach used to represent the central metabolism and L-carnitine biosynthesis subjected to long term adaptation to osmotic stress conditions allows the influence of the osmotic stress condition on fluxes to be quantified.

The model analysis shows that, after salt up-shift, the energy and fermentation pathways in the central metabolism undergo substantial rearrangement as they move towards an enhanced energy production. This is the case of the increase observed in the fermentative (V_1_, V_2_, V_10_, V_11_), anaplerotic (V_12_ and V_13_) and succinate (V_4_ and V_6_; see [10]) fluxes. This is further illustrated by the observation that the increase in the fermentation flux, V_11_ (from Pool_2_ to Pool_3_), is not connected with fluxes towards Pool_4_, representing the tricaboxylic acid intermediates and the synthesis of biomass. The values of the osmotic stress associated with these fluxes reflect these changes.

Pyruvate kinase (V_10_) and Pyruvate dehydrogenase (V_11_), which render pyruvate and acetyl-CoA, respectively, control energy yields during growth [10]. In fact, the increase in Pyruvate kinase activity is in good agreement with the observed increased glycerol consumption (V_1_; g_OS1_), suggesting that glycolytic rates increased as a consequence of stress towards fermentation (g_OS2_, g_OS8_, g_OS9_, g_OS10_, and g_OS14_). On the other hand, under anaerobiosis, acetyl-CoA is transformed to acetate (forming ATP) by phosphotransacetylase-acetate kinase (Pta-Ack) pathway (V_8_ in our model, which increases, see [27])

This would permit for increased energy synthesis to withstand the osmotic stress and would explain the increased rates of generation of fermentation products.

Furthermore, under anaerobic conditions, the TCA cycle (Pool_1_ and Pool_4_) provides biosynthetic precursors. In osmotic stress conditions, the activities of the controlling anaplerosis and gluconeogenesis enzymes isocytrate lyase, phosphoenol pyruvate carboxylase and phosphoenol pyruvate carboxykinase are altered [10]. This demonstrates the effect of long-term salt stress as regards cellular needs for anaplerotic intermediaries and energy (Pool_1_ and Pool_4_, reversibly connected with Pool_2_). These pathways allow the oxalacetate pool needed for biosynthesis to be replenished (Pool_1_ node). Taken together, the model results presented demonstrate the involvement of the biosynthetic pathways in the adaptation to osmotic conditions, since they are required for the production of cellular structural components. Greater fluxes in the central energy-producing and anaplerotic pathways have also been found in *C. glutamicum* when exposed to increased osmolarity [28]. Interestingly, the model outcome confirms the importance of acetate metabolism during long-term exposure to salt and during stress adaptation.

Further, in the transition phase, osmotic adaptations of *E. coli* metabolism translates into monotonic increases and decreases, with the exception of peptone and fumarate uptake into the cell which initially showed decreased concentrations.

Regarding the role of the osmoprotectant L-carnitine, we conclude that its catabolism is negatively influenced by the osmotic stress, while its synthesis and excretion is unaffected by any osmotic stress response mechanism.

## Supporting Information

Table S1 **Mean parameters and SD values of the selected solutions.**
(DOCX)

Table S2 **System parameters value for six solutions.**
(DOCX)
