# Supplementary material for: Modelling and Analysis of Central Metabolism Operating Regulatory Interactions in Salt Stress Conditions in a L-Carnitine Overproducing E. coli Strain
Source: PLoS One. 2012 Apr 13;7(4):e34533. doi: 10.1371/journal.pone.0034533 (PMC3326044; doi:10.1371/journal.pone.0034533)
Supplement: Table S1 — Mean parameters and SD values of the selected solutions. (DOCX) [file pone.0034533.s001.docx]

**SUPPORTING INFORMATION S1**

| **Parameter** | Mean value | SD | **Parameter** | Mean value | SD | **Parameter** | Mean value | SD |
| --- | --- | --- | --- | --- | --- | --- | --- | --- |
| **γ_1_** | 0.02 | 0 | **g_OS1_** | 0.32 | 0.09 | **g_910_** | 0.85 | 0.21 |
| **γ_2_** | 12.79 | 2.41 | **g_OS2_** | 1.12 | 0.27 | **g_107_** | 1.65 | 0.24 |
| **γ_3_** | 0.4 | 0.16 | **g_OS3_** | 0.23 | 0.06 | **g_118_** | 1.69 | 0.38 |
| **γ_4_** | 2.29 | 1 | **g_OS4_** | 0 | 0 | **g_1210_** | 0.09 | 0.04 |
| **γ_5_** | 3.17 | 1.41 | **g_OS5_** | 0.67 | 0.17 | **g_137_** | 0.26 | 0.09 |
| **γ_6_** | 7.52 | 1.35 | **g_OS6_** | -0.6 | 0.18 | **g_1410_** | 2.64 | 0.32 |
| **γ_7_** | 9.45 | 1.51 | **g_OS7_** | -1.65 | 0.18 | **g_158_** | 0.37 | 0.07 |
| **γ_8_** | 5.31 | 0.59 | **g_OS8_** | 1.61 | 0.27 | **g_1610_** | 0 | 0 |
| **γ_9_** | 2.21 | 1.05 | **g_OS9_** | 0.93 | 0.33 | **g_179_** | 0.35 | 0.05 |
| **γ_10_** | 10.56 | 3.74 | **g_OS10_** | 1.11 | 0.28 | **g_53_** | 1.15 | 0.31 |
| **γ_11_** | 3.52 | 0.35 | **g_OS11_** | -1.23 | 0.23 | **g_64_** | 0.12 | 0.03 |
| **γ_12_** | 1.41 | 0.58 | **g_OS12_** | 0.54 | 0.13 | **g_59_** | 0.48 | 0.11 |
| **γ_13_** | 3.11 | 1.69 | **g_OS13_** | 0 | 0 | **g_1814_** | 1.35 | 0.44 |
| **γ_14_** | 9.38 | 2.26 | **g_OS14_** | 1.15 | 0.37 | **g_47_** | -0.65 | 0.16 |
| **γ_15_** | 2.21 | 1.71 | **g_OS15_** | 0.44 | 0.15 | **K_delay_** | 0.19 | 0.06 |
| **γ_16_** | 0.87 | 0.37 | **g_OS16_** | -0.58 | 0.12 |  |  |  |
| **γ_17_** | 2.64 | 1.44 | **g_OS17_** | -0.11 | 0.04 |  |  |  |
| **γ_18_** | 3.37 | 0.71 | **g_OS18_** | -0.41 | 0.19 |  |  |  |

**Table S1. Mean parameters and SD values of the selected solutions.**
