# Supplementary material for: Modelling and Analysis of Central Metabolism Operating Regulatory Interactions in Salt Stress Conditions in a L-Carnitine Overproducing E. coli Strain
Source: PLoS One. 2012 Apr 13;7(4):e34533. doi: 10.1371/journal.pone.0034533 (PMC3326044; doi:10.1371/journal.pone.0034533)
Supplement: Table S2 — System parameters value for six solutions. (DOCX) [file pone.0034533.s002.docx]

**SUPPORTING INFORMATION S2**

|  | **Solution 1** | | **Solution 2** | | **Solution 3** | | **Solution 4** | | **Solution 5** | | **Solution 6** | |  |
| --- | --- | --- | --- | --- | --- | --- | --- | --- | --- | --- | --- | --- | --- |
| **γ_1_** | 0.02 | | 0.02 | | 0.02 | | 0.02 | | 0.02 | | 0.02 | |  |
| **γ_2_** | 9.47 | | 11.05 | | 11.83 | | 15.2 | | 13.62 | | 15.55 | |  |
| **γ_3_** | 0.23 | | 0.22 | | 0.63 | | 0.46 | | 0.39 | | 0.46 | |  |
| **γ_4_** | 2.62 | | 0.61 | | 2.74 | | 3.47 | | 1.7 | | 2.59 | |  |
| **γ_5_** | 1.87 | | 1.45 | | 3.08 | | 3.28 | | 4.01 | | 5.31 | |  |
| **γ_6_** | 6.08 | | 9.74 | | 6.82 | | 7.54 | | 6.57 | | 8.37 | |  |
| **γ_7_** | 7.24 | | 8.55 | | 9.26 | | 9.54 | | 10.46 | | 11.62 | |  |
| **γ_8_** | 4.75 | | 5.52 | | 5.59 | | 4.44 | | 6.02 | | 5.53 | |  |
| **γ_9_** | 1.6 | | 1.43 | | 2.19 | | 1.14 | | 3.02 | | 3.87 | |  |
| **γ_10_** | 4.73 | | 11.65 | | 9.29 | | 9.05 | | 15.45 | | 13.18 | |  |
| **γ_11_** | 3.45 | | 3.86 | | 3.57 | | 2.96 | | 3.37 | | 3.89 | |  |
| **γ_12_** | 0.49 | | 1.05 | | 1.85 | | 2.04 | | 1.29 | | 1.75 | |  |
| **γ_13_** | 0.53 | | 2.69 | | 3.68 | | 3.83 | | 2.33 | | 5.57 | |  |
| **γ_14_** | 9.11 | | 6.9 | | 7.3 | | 12.29 | | 8.82 | | 11.86 | |  |
| **γ_15_** | 0.14 | | 0.91 | | 1.84 | | 1.84 | | 4.11 | | 4.4 | |  |
| **γ_16_** | 0.21 | | 1.25 | | 1.03 | | 0.69 | | 0.92 | | 1.1 | |  |
| **γ_17_** | 0.01 | | 3.35 | | 3.53 | | 2.14 | | 2.81 | | 4.01 | |  |
| **γ_18_** | 3.1 | | 2.17 | | 4.12 | | 3.99 | | 3.33 | | 3.53 | |  |
| **g_910_** | 1.09 | | 0.5 | | 0.88 | | 0.8 | | 0.75 | | 1.06 | |  |
| **g_107_** | 2.08 | | 1.34 | | 1.6 | | 1.65 | | 1.57 | | 1.65 | |  |
| **g_118_** | 1.52 | | 2.42 | | 1.52 | | 1.73 | | 1.36 | | 1.6 | |  |
| **g_1210_** | 0.03 | | 0.13 | | 0.07 | | 0.11 | | 0.11 | | 0.08 | |  |
| **g_137_** | 0.12 | | 0.34 | | 0.26 | | 0.24 | | 0.24 | | 0.38 | |  |
| **g_1410_** | 3.1 | | 2.75 | | 2.51 | | 2.85 | | 2.33 | | 2.3 | |  |
| **g_158_** | 0.49 | | 0.27 | | 0.37 | | 0.35 | | 0.39 | | 0.33 | |  |
| **g_1610_** | 0 | | 0 | | 0 | | 0 | | 0 | | 0 | |  |
| **g_179_** | 0.28 | | 0.3 | | 0.38 | | 0.42 | | 0.33 | | 0.38 | |  |
| **g_53_** | 0.71 | | 1.44 | | 0.95 | | 0.99 | | 1.47 | | 1.34 | |  |
| **g_64_** | 0.17 | | 0.12 | | 0.12 | | 0.11 | | 0.09 | | 0.12 | |  |
| **g_59_** | 0.35 | | 0.45 | | 0.38 | | 0.48 | | 0.59 | | 0.63 | |  |
| **g_1814_** | 0.91 | | 0.92 | | 1.43 | | 1.12 | | 1.95 | | 1.77 | |  |
| **g_47_** | -0.64 | | -0.94 | | -0.57 | | -0.47 | | -0.7 | | -0.55 | |  |
| **K_delay_** | 0.09 | | 0.12 | | 0.21 | | 0.25 | | 0.23 | | 0.23 | |  |
|  | | **Solution 1** | | **Solution 2** | | **Solution 3** | | **Solution 4** | | **Solution 5** | | **Solution 6** | |
| **gos_1_** | | 0.39 | | 0.38 | | 0.18 | | 0.27 | | 0.4 | | 0.28 | |
| **gos_2_** | | 1.15 | | 0.92 | | 0.72 | | 1.5 | | 1.14 | | 1.29 | |
| **gos_3_** | | 0.34 | | 0.24 | | 0.18 | | 0.24 | | 0.18 | | 0.22 | |
| **gos_4_** | | 0 | | 0 | | 0 | | 0 | | 0 | | 0 | |
| **gos_5_** | | 0.58 | | 0.53 | | 0.74 | | 0.53 | | 0.67 | | 0.97 | |
| **gos_6_** | | -0.39 | | -0.47 | | -0.56 | | -0.65 | | -0.64 | | -0.9 | |
| **gos_7_** | | -1.92 | | -1.75 | | -1.62 | | -1.73 | | -1.46 | | -1.45 | |
| **gos_8_** | | 1.54 | | 1.77 | | 2.08 | | 1.36 | | 1.38 | | 1.55 | |
| **gos_9_** | | 1.4 | | 0.59 | | 0.92 | | 1.23 | | 0.6 | | 0.84 | |
| **gos_10_** | | 1.35 | | 0.74 | | 1.04 | | 1.52 | | 0.95 | | 1.09 | |
| **gos_11_** | | -1.04 | | -1.62 | | -1.38 | | -1.2 | | -1.11 | | -1.03 | |
| **gos_12_** | | 0.4 | | 0.48 | | 0.43 | | 0.56 | | 0.69 | | 0.71 | |
| **gos_13_** | | 0 | | 0 | | 0 | | 0 | | 0 | | 0 | |
| **gos_14_** | | 1.69 | | 0.95 | | 1.51 | | 0.79 | | 0.81 | | 1.13 | |
| **gos_15_** | | 0.17 | | 0.54 | | 0.58 | | 0.56 | | 0.39 | | 0.43 | |
| **gos_16_** | | -0.39 | | -0.61 | | -0.73 | | -0.59 | | -0.51 | | -0.64 | |
| **gos_17_** | | -0.14 | | -0.15 | | -0.07 | | -0.07 | | -0.13 | | -0.12 | |
| **gos_18_** | | -0.14 | | -0.34 | | -0.35 | | -0.37 | | -0.68 | | -0.57 | |
| **Pepto_0_** | | 0.55 | | 0.47 | | 0.44 | | 0.34 | | 0.38 | | 0.34 | |
| **Cro_0_** | | 2.15 | | 2.2 | | 1.18 | | 1.46 | | 1.62 | | 1.47 | |
| **Pool1_0_** | | 0.15 | | 0.08 | | 0.12 | | 0.1 | | 0.11 | | 0.09 | |
| **Pool2_0_** | | 0.02 | | 0.01 | | 0.01 | | 0.01 | | 0.01 | | 0.01 | |
| **Pool3_0_** | | 0.07 | | 0.06 | | 0.06 | | 0.07 | | 0.05 | | 0.06 | |
| **Pool4_0_** | | 0.69 | | 1.22 | | 1 | | 0.74 | | 0.93 | | 0.86 | |
| **Delay_pool1_1** | | 0.49 | | 1.13 | | 1.02 | | 0.64 | | 0.81 | | 0.75 | |
| **Delay_Pool2_1** | | 0.09 | | 0.38 | | 0.28 | | 0.3 | | 0.39 | | 0.44 | |
| **Delay_Pool3_1** | | 0.33 | | 0.21 | | 0.5 | | 0.38 | | 0.44 | | 0.48 | |
| **Delay_Pool4_1** | | 0.11 | | 0.19 | | 0.2 | | 0.18 | | 0.2 | | 0.18 | |
| **Delay_Pool1_2** | | 0.02 | | 0.09 | | 0.16 | | 0.13 | | 0.15 | | 0.11 | |
| **Delay_Pool2_2** | | 0.27 | | 0.16 | | 0.12 | | 0.16 | | 0.19 | | 0.21 | |
| **Delay_Pool3_2** | | 0.71 | | 0.64 | | 0.63 | | 0.67 | | 0.66 | | 0.61 | |
| **Delay_Pool4_2** | | 0.62 | | 0.27 | | 0.41 | | 0.51 | | 0.77 | | 0.67 | |

**Table S2. System parameters value for six solutions.**
